# Contemporary Medical Management of Primary Hyperparathyroidism: A Systematic Review

**DOI:** 10.3389/fendo.2017.00079

**Published:** 2017-04-20

**Authors:** Julius Simoni Leere, Jesper Karmisholt, Maciej Robaczyk, Peter Vestergaard

**Affiliations:** ^1^Department of Clinical Medicine, Aalborg University, Aalborg, Denmark; ^2^Department of Endocrinology, Aalborg University, Aalborg, Denmark

**Keywords:** primary hyperparathyroidism, parathyroid adenoma, medical treatment, drug therapy, bisphosphonates, cinacalcet

## Abstract

**Introduction:**

Primary hyperparathyroidism is increasingly an asymptomatic disease at diagnosis, but the recognized guidelines for management are based on evidence obtained from studies on patients with symptomatic disease, and surgery is not always indicated. Other patients are unable to undergo surgery, and thus a medical treatment is warranted. This systematic review provides an overview of the existing literature on contemporary pharmaceutical options available for the medical management of primary hyperparathyroidism.

**Methods:**

Databases of medical literature were searched for articles including terms for primary hyperparathyroidism and each of the included drugs. Data on s-calcium, s-parathyroid hormone, bone turnover markers, bone mineral density (BMD) and hard endpoints were extracted and tabulated, and level of evidence was determined. Changes in s-calcium were estimated and a meta-regression analysis was performed.

**Results:**

The 1,999 articles were screened for eligibility and 54 were included in the review. Weighted mean changes calculated for each drug in s-total calcium (mean change from baseline ± SEM) were pamidronate (0.31 ± 0.034 mmol/l); alendronate (0.07 ± 0.05 mmol/l); clodronate (0.20 ± 0.040 mmol/l); mixed bisphosphonates (0.16 ± 0.049 mmol/l); and cinacalcet (0.37 ± 0.013 mmol/l). The meta-analysis revealed a significant decrease of effect on s-calcium with time for the bisphosphonates (Coef. −0.049 ± 0.023, *p* = 0.035), while cinacalcet proved to maintain its effect on s-calcium over time. Bisphosphonates improved BMD while cinacalcet had no effect.

**Discussion:**

The included studies demonstrate advantages and drawbacks of the available pharmaceutical options that can prove helpful in the clinical setting. The great variation in how primary hyperparathyroidism is manifested requires that management should rely on an individual evaluation when counseling patients. Combining resorptive agents with calcimimetics could prove rewarding, but more studies are warranted.

## Introduction

Management of primary hyperparathyroidism is a topic of much debate. Surgery is today the only curative treatment option. Changes in the symptomatology and stage of disease at diagnosis have led to an increasing number of patients who do not fulfill criteria for surgery, but are managed with active surveillance (1). Some studies suggest that these patients over time might experience disease progression or develop complications such as osteoporosis, cardiovascular disease, or renal calculi (2–5). Other patients are unable to undergo surgery due to comorbidity or fear of complications. For these reasons, a medical alternative is highly warranted. In the past, several different pharmaceutical options have been investigated. Studies have reported trials utilizing estrogens and estrogen-related compounds (6), calcitonin (7), oral phosphate (8), strontium ranelate (9), somatostatin analogs (10, 11), vitamin-D and calcium supplementations (12). Some of these studies have shown minor effects on s-calcium-levels, bone mineral density (BMD), and other endpoints. Due to lack of effect, contraindications, and unwanted or unacceptable risks of drug-related adverse events, these drugs are today not considered realistic alternatives to surgery (13). The purpose of this review therefore was to describe the available literature on classes of drugs that today are considered options in the medical therapy of primary hyperparathyroidism where surgery for various reasons cannot be performed. This systematic review has its main focus on classes of antiresorptive medication (mainly bisphosphonates) and calcimimetics (cinacalcet).

## Materials and Methods

### Literature Search and Selection Strategy

Included articles were found searching Medline (1946–2016), Embase (1947–2016), and The Cochrane Library (inception–2016). Literature searches were conducted by an experienced librarian with input from the principal investigator. Searched terms included primary hyperparathyroidism, parathyroid adenoma, and each of the included pharmaceutical agents. Included drugs were all groups of bisphosphonates, isoflavones, denosumab, blosozumab, romosozumab, odanacatib, cinacalcet, etelcalcetide, telcalcetide, and velcalcetide with all available product names and synonyms. The final search was performed September 26th 2016, which thus is the inclusion limit. All published full-text articles in English including medical treatment of primary hyperparathyroidism as a main subject were considered eligible. *In vitro*/animal studies, abstracts, review articles, and case reports with less than five subjects were not eligible due to lack of data and comparability. A thorough search strategy can be found in the Supplementary Material, in the included search string. An electronic review protocol can be found at www.crd.york.ac.uk/prospero/, registration number: CRD42016053702. We followed the PRISMA guidelines (www.prisma-statement.org).

### Study Selection

Articles were initially screened independently by title and abstract by two authors (Julius Simoni Leere and Peter Vestergaard). Lists with the articles each of the authors had found eligible were compared and discussed. In case of disagreement between the authors, the full-text article was acquired for evaluation. The obtained full-text articles were excluded if they did not include medical treatment of primary hyperparathyroidism as a main subject, or did not include one or more of the drugs of interest (i.e., drugs used in the search strategy). Studies that lacked data or consistency in reporting on efficacy of the drugs, or did not fulfill the given criteria for eligibility mentioned previously in the search strategy were excluded. Change in s-total calcium (albumin adjusted) was a primary endpoint and necessary in statistical analysis. Studies that failed to report figures of either baseline or treatment levels of s-total calcium were excluded from the meta-calculations, but included in the tables for evaluation in the systematic review.

### Data Extraction and Handling

If feasible, the following data were extracted and tabulated from each included study: test-drug, dosage used, patient and study characteristics, treatment duration, s-calcium at baseline, s-calcium when treated, change in parathyroid hormone (PTH), change in urinary calcium excretion or calcium/creatinine ratio, change in bone turnover markers, change in BMD as measured by dual energy X-ray absorptiometry (DXA) in terms of a *T*/*Z*-score or percental change at different sites, and evaluation of hard endpoints (e.g., fractures, adverse events, stone formation, etc.). Differences and changes were described as mean ± SD/SEM where possible, alternatively as change in percent or simply as decrease/increase depending on the available data. Due to possible variation in arrays and machinery used for analysis of bone turnover markers and BMD, outcomes for BMD and bone turnover markers were classified as either increase/decrease or no change for each individual study. This minimized the influence from differences in arrays and DXA-scanners used by the different studies. Values reported were usually measured at either the last measurement performed before termination for long-term treatment studies, at nadir/peak-values for short-term/single infusion studies, or as otherwise stated (see Tables S1–S6 in the Supplementary Material).

### s-Calcium Levels

s-total calcium was handled differently from the other variables: if no actual values including SD/SEM were stated in the articles, values were obtained from graphs and figures if possible. If only ranges were provided, the mean was deducted by taking the average of the stated extremes.

### Level of Evidence

All included studies were graded according to the Oxford Centre for Evidence-Based Medicine 2011 Levels of Evidence (OCEBM levels) (14). The category applied was evaluation of treatment benefits. Level for each study was assessed initially depending on the type of study and could be graded up or down depending on study quality, precision in reporting, population size, and effect size. Randomized controlled trials (RCTs) were considered most reliable (grade 2), followed by non-randomized controlled cohorts/follow-up studies (grade 3), and finally observational uncontrolled studies, case series, and reports (grade 4). Levels were evaluated, discussed, and approved by two authors (Julius Simoni Leere and Peter Vestergaard). Study quality was evaluated for each report by exploring factors that could possibly introduce bias, e.g., randomization, blinding, withdrawals and dropouts, as well as applied methods of analysis and reporting. For each drug-group, a mean level of provided evidence was calculated (see Table 1).

**Table 1:** **Oxford Center for Evidence-Based Medicine Levels of Evidence**.

Study	Drug	OCEBM level
**Pamidronate**		
van Breukelen et al. (15)	Pamidronate	3
Jansson et al. (16)	Pamidronate	4
Ishimura et al. (17)	Pamidronate	4
Schmidli et al. (18)	Pamidronate	2
Ammann et al. (19)	Pamidronate	4
Jansson and Morgan (20)	Pamidronate	4
Phitayakorn and McHenry (21)	Pamidronate	4
**Mean level of evidence—pamidronate: 3.57**		
**Alendronate**		
LoCascio et al. (22)	Alendronate	4
Khan et al. (23)	Alendronate	2
Adami et al. (24)	Alendronate	4
Szymczak and Bohdanowicz-Pawlak (25)	Alendronate	3
Akbaba et al. (26)	Alendronate	2
Cesareo et al. (27)	Alendronate	3
Chow et al. (28)	Alendronate	2
Hassani et al. (29)	Alendronate	3
Khan et al. (30)	Alendronate	2
Makras et al. (31)	Alendronate	4
Parker et al. (32)	Alendronate	3
Rossini et al. (33)	Alendronate	3
**Mean level of evidence—alendronate: 2.92**		
**Clodronate**		
Adami et al. (34)	Clodronate	3
Shane et al. (35)	Clodronate	2
Douglas et al. (36)	Clodronate	4
Douglas et al. (37)	Clodronate	4
Fang et al. (38)	Clodronate	4
Hamdy et al. (39)	Clodronate	4
**Mean level of evidence—clodronate: 3.5**		
**Etidronate and other bisphosphponates**		
Horiuchi et al. (40)	Etidronate	2
Kaplan et al. (41)	Etidronate	4
Reasner et al. (42)	Risedronate	4
Tournis et al. (43)	Risedronate	3
Rossini et al. (44)	Neridronate	4
Segula et al. (45)	Mixed	4
Lee et al. (46)	Mixed	4
Vera et al. (47)	Mixed	4
Yeh et al. (48)	Mixed	4
**Mean level of evidence—etidronate and other bisphosphonates: 3.67**		
**Cinacalcet**		
Brardi et al. (49)	Cinacalcet	3
Cetani et al. (50)	Cinacalcet	4
Giusti et al. (51)	Cinacalcet	4
Filopanti et al. (52)	Cinacalcet	2
Marotta et al. (53)	Cinacalcet	4
Faggiano et al. (54)	Cinacalcet	4
Keutgen et al. (55)	Cinacalcet	4
Khan et al. (56)	Cinacalcet	2
Luque-Fernández et al. (57)	Cinacalcet	4
Moyes et al. (58)	Cinacalcet	4
Marcocci et al. (59)	Cinacalcet	4
Norman et al. (60)	Cinacalcet	4
Peacock et al. (61)	Cinacalcet	2
Peacock et al. (62)	Cinacalcet	4
Peacock et al. (63)	Cinacalcet	3
Sajid-Crockett et al. (64)	Cinacalcet	4
Shoback et al. (65)	Cinacalcet	2
Saponaro et al. (66)	Cinacalcet	4
Schwarz et al. (67)	Cinacalcet	4
**Mean level of evidence—cinacalcet: 3.47**		
**Ipriflavone**		
Mazzuoli et al. (68)	Ipriflavone	4

### Statistical Analysis

Data on s-total calcium from individual trials were pooled as applicable to calculate weighted mean change and SEM for each included drug. Weighting was applied to data taking into account population size and SD of each included project. Non-standard units on s-calcium reported in the articles were converted to standard international units (millimoles per liter). Normality of data was checked using QQ-plots in comparison with a standardized QQ-plot catalog. Meta-regression was performed using STATA 8.0 (Stata Corporation, College Station, TX, USA) to evaluate impact of baseline s-calcium levels, treatment duration, and applied drug on effect of treatments in terms of change in means s-calcium level over time. Time was transformed into a natural-logarithmic scale in the process of investigating time effect on bisphosphonate treatment. Where only grouped outcomes such as increase in BMD were possible to analyze, a binomial distribution was assumed, and the outcome tested against the assumed probability distribution.

## Results

### Study Selection

**Figure 1:**
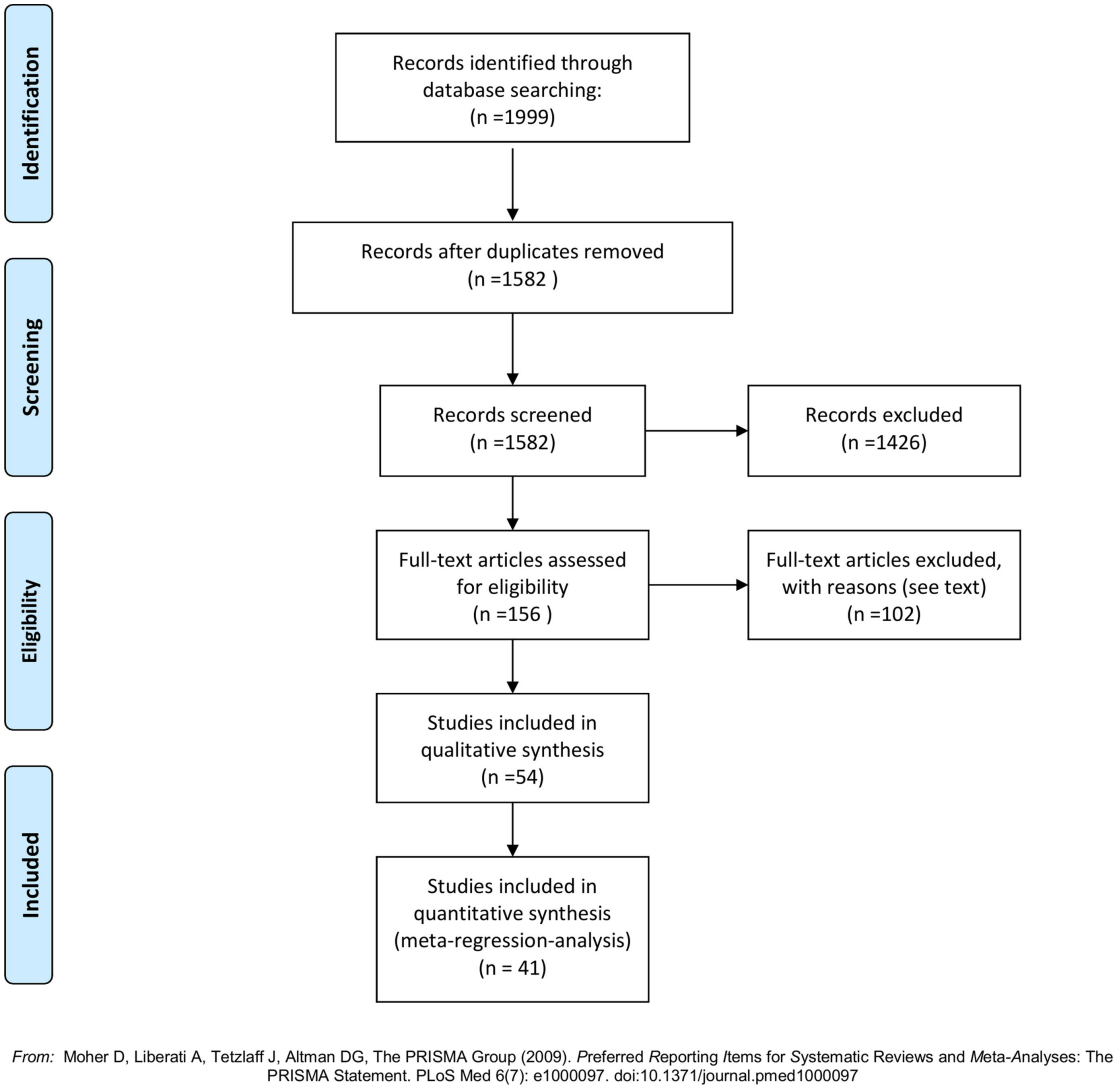
**PRISMA flowchart (80)**.

The literature search generated 1,999 articles: Embase (1,427), Medline (490), and Cochrane Library (82) (see flowchart, Figure 1). After removal of duplicates 1,582 articles remained. Of these 1,426 were deemed ineligible in the screening process of titles and abstracts due to following reasons: language other than English (118), *in vitro*/animal studies (5), review articles (117), and irrelevance for this review (1,186). In total, 156 articles were ordered from the Library of Aalborg University Hospital, to be assessed in full text. A total of 44 were published (conference) abstracts with no obtainable corresponding full-text article. A total of 41 were case reports with less than 5 included cases. One was a commentary on another article. One could not be delivered by the library (69). A total of 69 full-text articles were studied for eligibility. Fifteen were found ineligible; nine of these were focused on pharmaceutical agents outside the scope of this review [five vitamin-D supplementation and calcium (12, 70–73), two octreotide (10, 11), one hormone replacement therapy (6), one Strontium Ranelate (9)]; two were review articles (74, 75); two had no data on effect of the included drugs (76, 77); one had a main focus other than medical treatment of primary hyperparathyroidism (78); and one was found incomparable to the remaining studies due to incoherent reporting and few subjects (79). Fifty-four articles were thus included in this review. The articles were distributed between drug-classes as follows: 7 pamidronate, 12 alendronate, 6 clodronate, 2 etidronate, 2 risedronate, 1 neridronate, 4 mixed bisphosphonates, 19 cinacalcet, and 1 examining ipriflavone. See Tables S1–S6 in Supplementary Material for results of the individual reports. Forty studies provided sufficient data (figures on s-calcium including SD) to be included in the meta-regression on treatment effect on s-calcium levels over time, and the calculations of weighted means in change of s-calcium. The distribution of studies included in the meta-regression was as follows: 7 pamidronate, 9 alendronate, 3 clodronate, 1 etidronate, 1 risedronate, 3 mixed bisphosphonates, and 16 cinacalcet. Six of these studies provided data by means of graphs and figures (16, 18, 28, 30, 32, 56) and two (17, 58) reported only in terms of range, and values therefore were acquired as previously described.

### Pamidronate

The seven studies concerning management with pamidronate (15–21) were all studies of short-term treatment (see Table S1 in Supplementary Material). Duration of treatment ranged from a single infusion to a few weeks, and follow-up was correspondingly short. Mean level of evidence was 3.57. All studies report a significant reduction of s-calcium within days of treatment. Weighted change of mean in s-total calcium was (0.31 ± 0.034 mmol/l). Five studies report a transient rise in s-PTH, which decreased again as s-calcium returned toward baseline. Similarly, bone turnover decreased as measured by urinary calcium excretion and lowering of biochemical markers of bone resorption and formation (p-alkaline phosphatase; urinary hydroxyproline; p-osteocalcin). Due to the limited time-frame of performed studies, no measures of change in BMD were available. No serious drug-related adverse events were reported.

### Alendronate

Twelve studies reported on the use of alendronate (22–33) (see Table S2 in Supplementary Material). Range of treatment duration was 5 days to 2 years, with nine reports of ≥48 weeks. OCEBM Level of Evidence was on average 2.92. s-Total calcium decreased on average marginally (0.07 ± 0.05 mmol/l)—this figure is based on data from nine studies [two short-term (22, 24) and seven long-term studies (26–32)]. However, it is worth noticing that several of the studies reporting on long-term treatment found an initial significant decrease in s-calcium-levels, which lasted around 6 months, but then over time rose toward baseline (25, 26, 28, 32, 33). A corresponding pattern could be seen in PTH levels, which rose in the majority of studies. Of the three short-term studies (≤2 months) in the alendronate group, two found a decrease similar to that achieved in the pamidronate group (22, 24) whereas one found no change (31). Bone turnover decreased in all studies reporting on the issue in terms of biochemical turnover markers. Urinary calcium excretion was reported to either decrease or remain unchanged in eight studies whereas two studies found an insignificant increase. All nine long-term studies report on increases in BMD at all sites measure (except one study that finds an insignificant decrease in BMD in distal radius, but increase at all other sites). BMD-gains are reported to be more pronounced in bone rich in trabecular tissue (lumbar spine and hip/femoral neck) compared to compartments richer in cortical tissue (distal radius). The finding of an increase in BMD in all nine studies was significant as a decline is usually expected with age (*p* < 0.01 by binomial distribution assuming a likelihood of 5% for an increase in BMD).

### Clodronate

Clodronate was evaluated in a total of six studies (34–39) of which only three (35, 37, 39) had data valid for the calculation of an average decrease in s-total calcium of (0.20 ± 0.040 mmol/l) (see Table S3 in Supplementary Material). Duration of treatment was 2 weeks to 3 years. Short-term treatment was associated with a decrease in s-calcium in all five studies. Among these, the decrease was statistically significant in three. The only long-term study in the clodronate group (38) did not report figures on s-total calcium changes. Three studies report PTH increases, one finds no change and one report a slight decrease. All five studies describing changes in u-calcium changes find a treatment-related decrease. The same five studies report decreases in urinary hydroxyproline levels indicating a lowered bone resorption. Alkaline phosphatase is reported with some controversy between studies: three finds a slight decrease, two finds an increase (insignificant), and one finds no change. None of the studies report on changes in BMD. One study finds an increased rate of renal loss of function as well as a lower New York Heart Association Functional Classification (NYHA) and American Society of Anesthesiologists physical status classification (ASA) scores in a high morbidity patient-group compared to a group treated surgically; however, it remains unclear whether this can be related to the treatment with clodronate (38). Evidence level was on average 3.5.

### Mixed bisphosphonates

The articles concerning etidronate (2) (40, 41), risedronate (2) (42, 43), neridronate (1) (44), and mixed bisphosphonates (4) (45–48) were, due to the limited number of studies, pooled into one group called mixed bisphosphonates (see Table S4 in Supplementary Material). The articles reporting on effects of etidronate, risedronate, and neridronate used one drug only for all included subjects, whereas the articles on mixed bisphosphonates used a variety of different bisphosphonates within the individual study. One article on etidronate (40), one on risedronate (42), and three reporting on mixed bisphosphonates (45–47) provided data to the calculation of an average decrease in s-calcium of (0.16 ± 0.049 mmol/l). Treatment duration varied from a single dose treatment to 5 years. The OCEBM Level of Evidence in this pooled group was 3.67.

The five studies in this heterogeneous group reporting on a single bisphosphonates describe variable decreases in s-calcium and a simultaneous rise in PTH levels. Several articles thus described a pattern similar to what was observed in the articles of the alendronate group with an initial effect and later reversal (41, 44). Bone turnover markers [alkaline phosphatase, collagen type 1 cross-linked C-telopeptide (CTX), and osteocalcin] and urinary calcium excretion all decreased. The studies reporting on BMD-changes, demonstrated increases over time, and as in the other bisphosphonate groups this was markedly better at trabecular sites. One study concerning risedronate reported in addition to aBMD on a peripheral quantitative computed tomography analysis to evaluate the volumetric BMD, and found only very limited change from baseline (+0.24% at a trabecular site and −0.26% at a cortical site) (43).

Four articles describe cohorts treated with a range of bisphosphonates. Three long-term treatment regimens (45, 47, 48), and one short term (46). The short-term study describes the use of bisphosphonates in pretreatment of patients undergoing parathyroidectomy and claims that bisphosphonate treatment can prevent development of hungry bones syndrome [although the number of patients treated is very limited (*n* = 6)]. The three long-term studies have large populations included, but the dropout rate, especially in Ref (48). is high, leaving a risk of adherence bias. Only two out of the three (45, 47) reports on s-calcium which is unchanged in one and decreases in the other during treatment, PTH, CTX, and alkaline phosphatase are reported to decline. Vera et al. (47) reports a small and insignificant decline in BMD, whereas Yeh et al. and Segula et al. (45, 48) in accordance with most other studies report an increase at spine and hip. The prior study also reports a markedly increased risk of fractures compared to patients treated surgically (PTX) as well as controls, but the bisphosphonate subgroup also did have a much lower BMD at baseline compared to the other two groups. Segula et al. (45) reports no change in fragility fracture-rate when treated compared to when not treated with bisphosphonates.

### Cinacalcet

Cinacalcet was tested in 19 studies (49–67), 16 of which data on s-total calcium were pooled to estimate a weighted mean decrease of (0.37 ± 0.013 mmol/l) (see Table S5 in Supplementary Material). The mean level of evidence for the whole group was 3.47. Duration of the studies ranged from 15 days to 44 months. s-calcium decreased significantly and remained so in all 19 articles throughout the whole spectrum of treatment periods. This decrease was reported in ionized calcium as well (50–52, 64) and was also proved to be present in hereditary forms of primary hyperparathyroidism (51, 52, 58, 66).

Cinacalcet’s ability to lower PTH was intensely studied. In 18 studies, PTH is reported to decrease (significantly in 15) while one saw no change. The effect is reported to be smaller than that on s-calcium, and normalization of mean levels rarely occur. A few studies described how levels varied in relation to time since ingestion, and nadir was reported to be 2–4 h post dose, where-after it rose markedly and peaked pre-dose (65). There seems to be a tendency toward a lowering of urinary calcium excretion, with insignificant decreases reported in six studies, one showed significant decrease, two reported no change, and one an insignificant increase. The effect on bone turnover as measured by biomarkers appears to be diverse with two articles reporting a decline, four finding no change, and four indicating an increase. This is in accordance with no change on average (*p* = 0.79 by binomial distribution assuming a 33% likelihood of decline). Only a few of the studies performed measurements of BMD. Two of those studies report a marginal increase, two a decrease and five no change. This points at no overall change in BMD (*p* = 0.39 by binomial distribution assuming a 33% likelihood of decline). One study combined cinacalcet with alendronate and found the increase in BMD similar to that of the bisphosphonate therapy alone, but also maintained a significant decrease in s-calcium, u-calcium excretion, s-PTH, and bone turnover markers (alkaline phosphatase) throughout the year the study lasted (54). Like in the bisphosphonate groups the reporting of hard endpoints was very sparse. One study demonstrated a decrease in formation of nephrolithiasis and diameter of stones when treated with cinacalcet compared to standard treatment (49). Nine studies reported of drug-related adverse events. Two articles reported on HRQoL (health-related quality of life); one found an increase in 50% of patients with intractable primary hyperparathyroidism with the introduction of cinacalcet (59), the other found no difference compared to placebo (56). The most common side effects by far were nausea and gastrointestinal discomfort, myalgia and hypocalcemia (non-fatal).

### Meta-Regression on Treatment Effects on s-Total Calcium over Time

One of the purposes with this review was to evaluate the impact of time on treatment efficacy in terms of effect on s-calcium. This was done using a meta-regression-analysis. In addition to treatment duration we investigated whether baseline s-total calcium levels and drug class had an impact on the observed change. When performing the analysis on data on bisphosphonates with time being logarithmic transformed, it becomes evident that an increase in pretreatment s-calcium levels is positively correlated with the degree of decrease in s-calcium during treatment (Coef. 0.49 ± 0.16, *p* = 0.002). Treatment duration is negatively correlated to change of s-calcium (Coef. −0.049 ± 0.023, *p* = 0.035), and class of bisphosphonate utilized has no impact on effect (*p* = 0.227). If time is not transformed, treatment duration becomes insignificant (Coef. −0.00014 ± 0.00008, *p* = 0.075), and drug class becomes significant (*p* < 0.000), possibly due to the variation in treatment duration described between the different classes of bisphosphonate.

**Figure 2:**
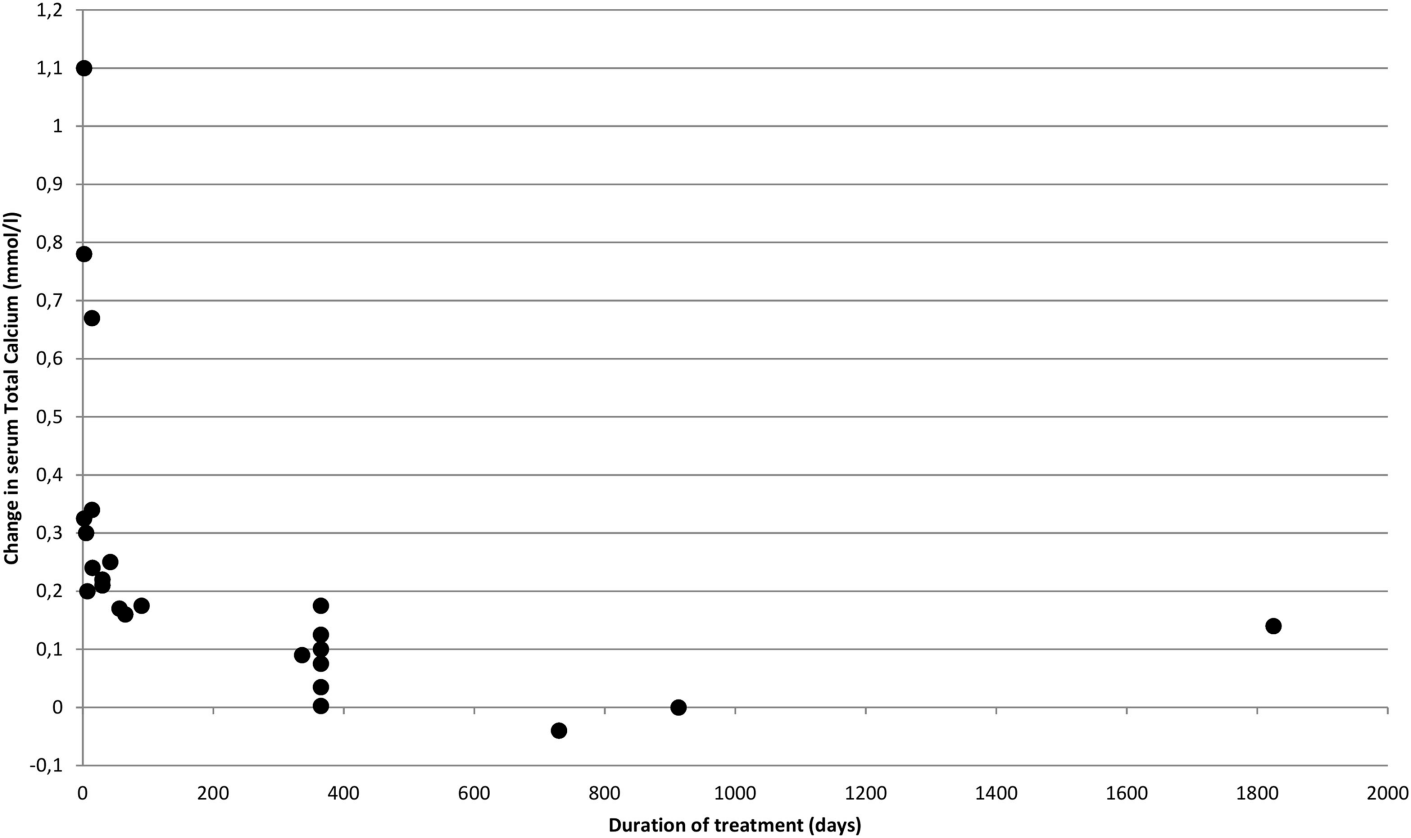
**Effect of bisphosphonates on s-calcium over time**.

**Figure 3:**
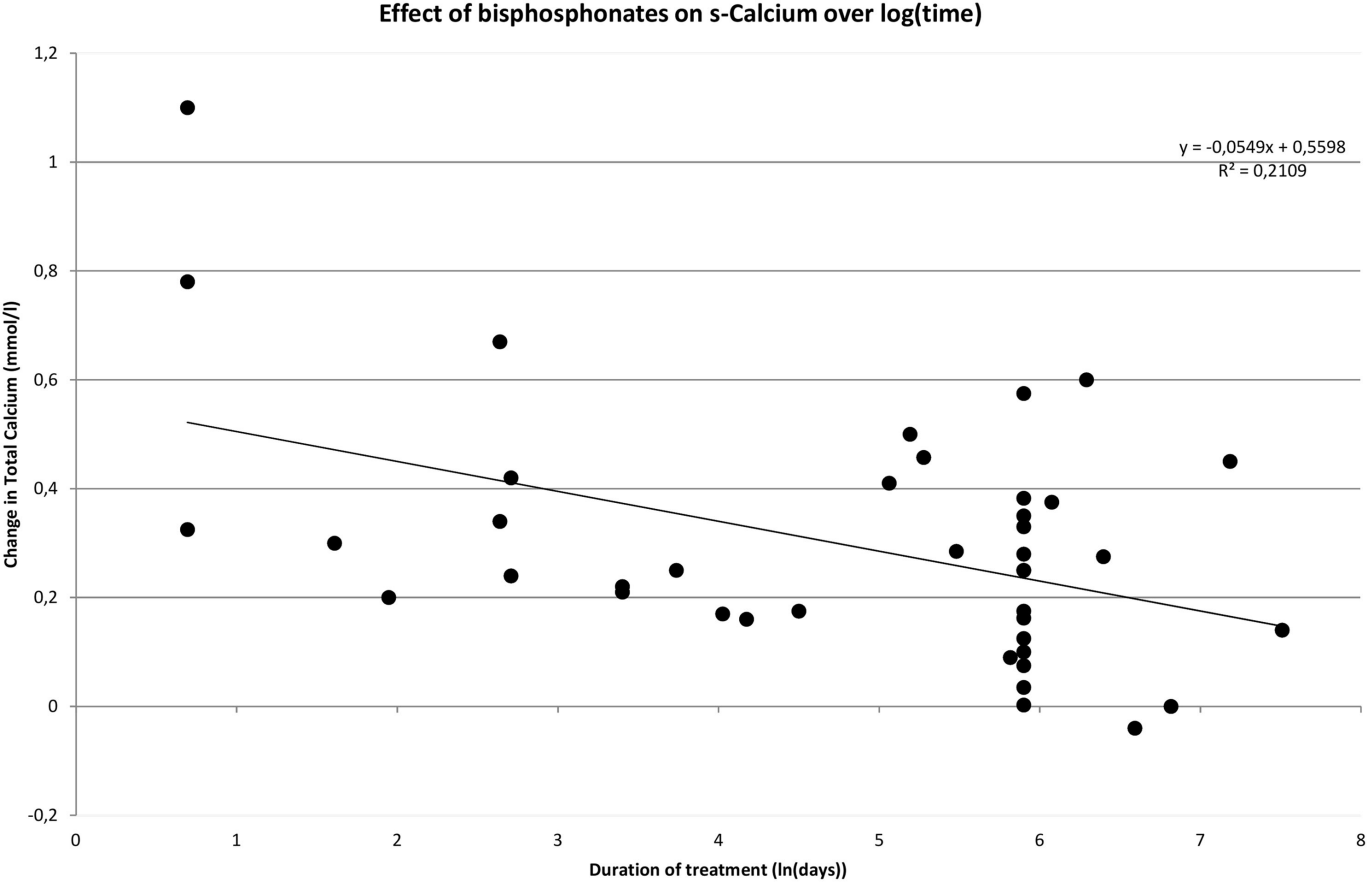
**Effect of bisphosphonates over time logarithmically transformed**.

Figure 2 shows how rapidly serum calcium decreases upon initiation of bisphosphonate. However, the decrease diminishes over time. This pattern is confirmed in Figure 3 where time has been transformed on a logarithmic scale, and a trend line clearly indicates a decrease in effect over time. The distribution of short- and long-term regimens between classes of bisphosphonates (e.g., all pamidronate studies had a short duration vs. most alendronate studies being long term), do thus not appear to make a clear difference in effect. Hence, the much smaller decrease in mean s-total calcium as reported previously for alendronate vs. pamidronate (0.066 ± 0.05 vs. 0.31 ± 0.034) may be attributed to a difference in time-span in the reported studies.

**Figure 4:**
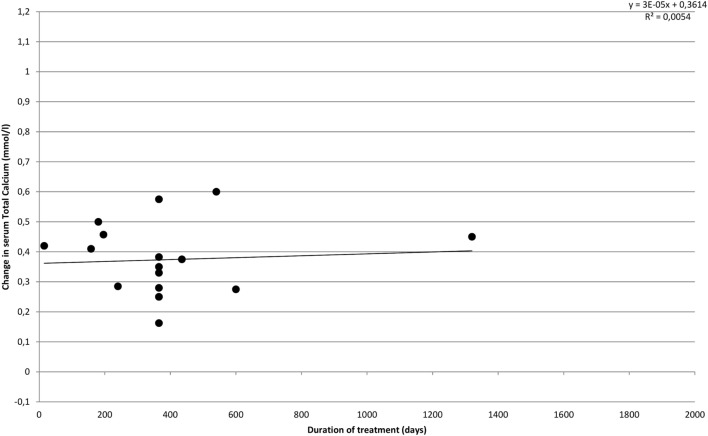
**Effect of cinacalcet on s-calcium over time**.

Figure 4 illustrates the effect of cinacalcet on s-total calcium over time. In accordance with results reported in the individual included studies, we find that the effect does not appear to change over time, but s-calcium remains lowered compared to baseline values throughout the treatment period.

## Discussion

In this systematic review and meta-analysis, we find a significant decrease of effect on plasma calcium with time for the bisphosphonates, while cinacalcet seems to maintain its effect on plasma calcium over time. Cinacalcet did not seem to have major effects on BMD and bone turnover, while long-term treatment with bisphosphonates seemed to increase BMD.

This illustrates the strengths and drawbacks of the pharmaceutical agents that are available at present. Bisphosphonates lower the levels, s-calcium levels, transiently and can be utilized in acute treatment, but are inefficient in regulating calcium-levels in the long term. As described by the meta-regression, baseline calcium-levels are positively correlated with the effect of bisphosphonate treatment on s-calcium, whereas the specific class of bisphosphonates appears to be less important when “adjusting” for distribution of treatment duration between drugs by transforming time to a logarithmic scale. The rebound of calcium-levels is probably due to the reported concomitant surge in s-PTH, which among other possible mechanisms will affect renal reabsorption of calcium, elevating s-calcium. On the other hand bisphosphonates lower bone turnover as shown by biochemical markers and significantly increase BMD especially at trabecular sites. Data on hard endpoints such as fracture-incidence are very sparse.

Cinacalcet proved to be efficient in lowering and normalizing s-calcium throughout the whole specter of studied disease severity, and, contrary to bisphosphonates, did not lose its calcium-lowering capability over time. It simultaneously lowers s-PTH, albeit to a less significant degree. It lowers u-calcium excretion minutely, possibly having an attenuating effect on renal stone disease. Bone turnover appears unchanged, and BMD does not improve. Hard endpoints other than brief reports on adverse events have rarely been evaluated. Due to the short period of time, cinacalcet has been available, there are only very limited data on long-term effects. This includes possible drug-related adverse events, and the future studies will need to focus on cinacalcet’s impact on bone turnover and hard endpoints.

A recent meta-analysis (81) finds only very limited evidence on clinical benefits of surgery vs. active surveillance in patients with mild primary hyperparathyroidism. The authors reviewed variables such as changes in BMD, quality of life measures, and hard endpoints such as kidney stones, fractures, and cardiovascular events. No convincing evidence of improvement by PTX was found for any of these endpoints, except a single RCT showing a minute effect on BMD. Although follow-up was short (max. 5 years), and the number of studies and patients relatively low, this demonstrates an important problem in the management of primary hyperparathyroidism of today: many patients are diagnosed in an asymptomatic stage of the disease, and all the evidence we apply in our counseling to them is based on studies involving cohorts at much higher risk. Our study involved patients of all disease severities and is therefore not directly comparable to the group investigated by Singh Ospina et al. (81). Furthermore, we studied the effects of drug intervention whereas Singh Ospina et al. (81) compared conservative management to surgical treatment. The clinical implications of our study for the group of asymptomatic patients, however, do appear relevant. If active surveillance proves to be equal to surgery on a long-term basis for this large group of patients, the addition of, e.g., bisphosphonates could turn out beneficial in treating and preventing the development of osteoporosis in this group of patients as an alternative to surgery. More studies on this issue and a possible medical alternative to surgery therefore seem warranted.

Only one article (54) had a main focus on a combined treatment (alendronate and cinacalcet), though the thought of utilizing the bone-strengthening effects of an antiresorptive agent in combination with the hormone- and calcium-regulating features of cinacalcet appears promising. Also noteworthy was the fact that no trials had been conducted testing the effects of denosumab or zoledronic acid in primary hyperparathyroidism. New drugs under evaluation such as etelcalcitide could also turn out to be valuable additives to the current options in the future treatment of primary hyperparathyroidism.

This study has its limitations due to relatively few reports on each drug class, the variability in duration of treatment reported from one drug to the other, and in low general level of evidence (small study-populations, few RCTs, inconsistency in reporting results, risk of bias, and confounders of the included studies). Usually serum calcium is tightly regulated and the variation is small. Influence from various assays used by the studies cannot be excluded, but must be regarded as minor. The calculated mean s-calcium levels and SDs were in one case based on a ranges (17) and in seven cases based on figures obtained from graphs (16, 18, 28, 30, 32, 53, 56), giving a risk of minor deviations from the exact values due to reading/printing imprecisions. Such deviations would however only have low impact on the meta-regression-analysis and calculated means.

In conclusion, bisphosphonates seem to have a short-lived (<6 months) effect on plasma calcium, but a positive effect on BMD. By contrast, cinacalcet maintains a long-term effect on plasma calcium, but does not improve BMD.

## Author Contributions

JL and PV designed the study and performed analysis. JL in cooperation with the library acquired the literature and drafted the work. JL, JK, MR, and PV all contributed to interpretation, revising, and approved the final version to be published. All authors agreed to be accountable for all aspects of the work.

## Conflict of Interest Statement

The authors declare that the research was conducted in the absence of any commercial or financial relationships that could be construed as a potential conflict of interest.
