# Corrigendum: Contemporary Medical Management of Primary Hyperparathyroidism: A Systematic Review

**DOI:** 10.3389/fendo.2017.00172

**Published:** 2017-07-20

**Authors:** Julius Simoni Leere, Jesper Karmisholt, Maciej Robaczyk, Peter Vestergaard

**Affiliations:** ^1^Department of Clinical Medicine, Aalborg University, Aalborg, Denmark; ^2^Department of Endocrinology, Aalborg University, Aalborg, Denmark

**Keywords:** primary hyperparathyroidism, parathyroid adenoma, medical treatment, drug therapy, bisphosphonates, cinacalcet

## Missing Supplementary Information

In the original article, there was a mistake in the published supplementary material. The literature search string as mentioned in the section “Literature Search and Selection Strategy” never appeared in the published material. The search string as applied in Medline appears below. The authors apologize for this error and state that this does not change the scientific conclusions of the article in any way.

Ovid Technologies, Inc. Email Service

Database: Epub Ahead of Print, In-Process & Other Non-Indexed Citations, Ovid MEDLINE(R) Daily and Ovid MEDLINE(R)<1946 to Present>

Search Strategy:

1 Hyperparathyroidism, Primary/dt [Drug Therapy] (99)

2 Hyperparathyroidism, Primary/(2211)

3 Parathyroid adenom*.mp. (4188)

4 (Primary adj2 Hyperparathyr*).mp. (9043)

5 2 or 3 or 4 (11256)

6 exp Diphosphonates/(22931)

7 bisphosphonate*.mp. (14211)

8 diphosphonat*.mp. (17321)

9 bisphosphonic*.mp. (152)

10 clodron*.mp. (2326)

11 alendron*.mp. (4714)

12 etidron*.mp. (3123)

13 ibandron*.mp. (1063)

14 incadron*.mp. (78)

15 medron*.mp. (4405)

16 minodron*.mp. (104)

17 neridron*.mp. (94)

18 olpadron*.mp. (77)

19 pamidron*.mp. (2920)

20 risedron*.mp. (1775)

21 tiludron*.mp. (156)

22 zoledron*.mp. (4145)

23 cinacalcet.mp. (1071)

24 Cinacalcet Hydrochloride/(735)

25 "amg 073".mp. (24)

26 amg073.mp. (0)

27 krn 1493.mp. (3)

28 krn1493.mp. (4)

29 mimpara.mp. (20)

30 parareg.mp. (0)

31 regpara.mp. (1)

32 sensipar.mp. (30)

33 exp Isoflavones/(15928)

34 ipriflavone.mp. (268)

35 Denosumab/(925)

36 denosumab.mp. (1712)

37 amg 162.mp. (33)

38 amg162.mp. (3)

39 amgiva.mp. (0)

40 prolia.mp. (32)

41 xgeva.mp. (16)

42 blosozumab.mp. (14)

43 ly 2541546.mp. (0)

44 ly2541546.mp. (1)

45 romosozumab.mp. (45)

46 amg 785.mp. (27)

47 amg785.mp. (0)

48 cdp 7851.mp. (0)

49 cdp7851.mp. (3)

50 odanacatib.mp. (156)

51 ("mk 0822" or mk0822 or mk822 or mk 822).mp. (5)

52 etelcalcetide.mp. (5)

53 (amg 416 or amg416).mp. (12)

54 (kai 4169 or kai4169).mp. (1)

55 (ono 5163 or ono5163).mp. (0)

56 telcalcetide.mp. (0)

57 velcalcetide.mp. (4)

58 or/6-57 (49081)

59 5 and 58 (459)

60 1 or 59 (491)

## Conflict of Interest Statement

The authors declare that the research was conducted in the absence of any commercial or financial relationships that could be construed as a potential conflict of interest.

