# Supplementary material for: Contemporary Medical Management of Primary Hyperparathyroidism: A Systematic Review
Source: Front Endocrinol (Lausanne). 2017 Apr 20;8:79. doi: 10.3389/fendo.2017.00079 (PMC5397399; doi:10.3389/fendo.2017.00079)
Supplement: Supplementary file 1 [file Data_Sheet_1.docx]

**Data tables:**

Variables are given in mean values, and compared to baseline unless otherwise stated.

In several of the long-term treatment studies, there is a significant drop-out of subjects, and as such there is a risk of the presented figures being influenced by adherence bias. Analysis in the included studies is generally based on available data without imputation of missing values. Differences and changes in mean values are given for the treated period of time, usually measured at either the latest measurement before termination for long -term treatment studies, at nadir/peak-values for short term/single infusion studies, or as otherwise stated.

Table 1: Pamidronate.

| Drug/ dose, mg/d | Patient/ study character | Treat-ment duration | Baseline s-calcium¤, mean (mmol/l) | Mean s-calcium, treated (mmol/l) | Mean change PTH (ng/L) | Mean change u-calc. (calcium :creatinine) | Bone turnover markers | Mean change BMD | Hard end-points | Study/ ref. | OCEBM |
| --- | --- | --- | --- | --- | --- | --- | --- | --- | --- | --- | --- |
| Pamidro-nate, average between 300-1200mg/d, Oral solution | 47 patients, all treated, 27 with hyper-calcaemia (8phpt), 20controls (7 Osteo -porosis, 13 Paget’s disease ) | 2wks | Phpt: 2.83±0.11  Controls (Paget’s): 2.32±0.02 | Nadir: Phpt: 2.49 ±0.10*  Controls (Paget’s): 2.17±0.03* | N/A | 24h Ca/crea:  Phpt: * 0.76 ±0.11 to 0.29±0.08 Controls (Paget’s):* 0.50±0.07 to 0.20±0.05 | 24h Uhp: creatinine Phpt: ↓* Controls (Paget’s): ↓* | N/A | N/A | (van Breukelen et al., 1982) | 3 |
| Pamidro-nate, single infusion 15-60 mg | 9 pts, all treated | 2 days | Total calcium: 3.13±0.28 Ionized: 1.61±0.13 | Nadir: After 7 days 2.35±0.2* ^(from figure)^ | Tran-sient rise. | N/A | N/A | N/A | N/A | (S Jansson, Tisell, Lindstedt, & Lundberg, 1991) | 4 |
| Pamidronate, 30mg, IV, once | 5 pts, all treated | 14 days | Tot.calcium3.23±0.33 (R:2.9-3.65) | Range for nadir: 2.35-2.77 | Tran-sient rise. | Spot Ca/crea:↓ (transient decrease) | Uhp:↓ ALP:↓ OC:↓ | N/A | N/A | (Ishimura et al., 1993) | 4 |
| Pamidronate, 30mg infusion/placebo | 10 pts, RCT, crossover | 7 days | 2.69±0.07 ^(from figure)^ | Nadir: Pamidr.:*2.49±0.04 Placebo: 2.72±0.06 | Pami: *^vs^ 448±48 Plac: 392±42 | Ca/crea: Pamidr.:*^vs^ 0.49±0.07 Placebo: 0.78±0.15 | ALP:~ Pamid.: 98.8±7.2 Placebo: 94.9±6.7 U/l | N/A | NS change blood pressure, muscle strength, symptoms or cognitive function | (Schmidli, Wilson, Espiner, Richards, & Donald, 1990) | 2 |
| Pami-dronate, 60-90mg n=23/ clo-dronate 600 mg, n=2 IV once | 25 elderly pts + 9 untreated controls | 15 days | 2.73±0.03 | 2.49 ±0.03* | 13.9±2.8 to 27.1±10.1* pmol/l | Ca/crea 0.62±0.09 to 0.34±0.11* mmol/mmol | N/A | N/A | Signi-ficant change in FIM, from baseline and com-pared to controls.* | (Ammann, Herter-Clavel, Lubrano, & Rizzoli, 2003) | 4 |
| Pami-dronate, 30-45mg, once | 20 pts, all treated 30 days+ prior to PTX | 30 days | 2.84±1.49 | Nadir (6 days post infusion): 2.63± 0.39* Ion: 1.44± 0.035* Post PTX: 2.16± 0.035* Ion: 1.19± 0.028* | Pre: 107± 35.0 Pami: (max) 173± 83.0* PTX: (nadir) 12.6± 9.1 ng/l | Excr: mmol/24h: (n = 6) Pre: 5.8 Post: Pamíd: 2.6 PTX: 1.8 | 30 days after infusion ALP: Pami: ↓* PTX:↓* OC: Pami:↓~ PTX:↓* | N/A | No serious adverse events | (Svante Jansson & Morgan, 2004) | 4 |
| Pamidronate 60 to 90 mg IV / 4 mg zoledronic acid once combined with fluid 16 ± 6 L and furo-semide 20 to 40 mg | 8 patients with hyperparathyroid crisis. Retrospect. | Acute treatment | 4.05 ± 0.4 | 2.95 ± 0.4* | N/A | N/A | N/A | N/A | Correction of ECG (n=1) and mental status | (Phitayakorn & McHenry, 2008) | 4 |
| ¤: albumin adjusted calcium unless stated otherwise; *p<0.05, significance compared to baseline;~:non-significant vs baseline; *^vs^:p<0.05 vs control-group; N/A: not available; ALP: p-alkaline phosphatase; Uhp: urinary hydroxyproline; OC: Osteocalcin; FIM: functional independence measure; ↑:denotes an increase; ↓: denotes a decrease; ↔: denotes no change. | | | | | | | | | | | |

Table 2: Alendronate

| Drug/dose, mg/day | Patient/ study character | Length of treat-ment. | Baseline s-calcium¤, (mmol/l) | Mean s-calcium, treated (mmol/l) | Mean change PTH (ng/L) | Mean change u-calc. (calcium :creatinine) | Bone turnover markers | Change BMD | Hard end-points | Study/ ref. | OCEBM |
| --- | --- | --- | --- | --- | --- | --- | --- | --- | --- | --- | --- |
| Alendronate 2.5mg IV Once | 12 pts, all treated | 5 days | 2.90±0.22 | 2.6±0.15* Rose again after few days | 17.5±6.38 to 22.8±8.8* pmol/l | Spot urine Ca/crea: 0.27±0.11 to 0.20 ± 0.08 | ALP: (-3.8%)~ Uhp: (-31%)* | N/A | N/A | (L. V. et al., 1994) | 4 |
| Alendronate 10 mg/day PO /Placebo | 9 men, RCT, cross-over | 1 year | Tot. calc.: 2.66 Ion: 1.39 | Tot. calc:~ 2.57 ^(-3.4%± 1.59%)^ Ion: 1.33 | Alendr:~ +35.1% ±15.36% Placebo: ↔ | -15.28% ~ | Alendr: ALP: * -46.6% ±9.57%NTX: * -60.6% ±27.7%~ Placebo: All ↔ | LS:* Alendr: +4.4% T-Hip:* +2.95% DR:~ +2.13% Placeb: All ↔ | N/A | (A.A., J.P., A., S.J., & T.I., 2009) | 2 |
| Alendronate 5 mg, IV Once. | 6 pts all treated | 56 days | 2.8 ±0.125 | 2.63±0.13* | No change | N/A | 2wk to nadir Uhp: 21.8±9 to 9.4* ALP:↓* | N/A | N/A | (Adami et al., 1994) | 4 |
| Alendronate 70 mg/wk PO vs PTX. | 63 pts, 30 PTX, 33Alend. 50 healthy controls | 12 mths | N/A | Alendr. No significant change. PTX: Normal-ization after 12 months. | Alendr.:~ 149.8± 62.4 to 163.6 ± 68.4pg/l PTX:* 238.9± 216.6 to 46.5±24.0 pg/l | Alendr: No significant change. PTX: Normal-ization after 12 months. | Alendr: OC:↓~£ ICTP: ↓~ ALP:↓* PTX: OC:↓↓* ICTP:↓* ALP:↓*  OPG/ RANKL-ratio is corrected in both PTX and alendr. after 12 months | Alendr: LS:* 3.4%± 5.4% FN:* 1.8%± 3.8% FUD:* 4.3%± 7.9% PTX: LS:* 8.9%± 12.3% FN:* 6.9%± 13.8% FUD:* 2.7% ±8.8% | N/A | (Szymczak & Bohdanowicz-Pawlak, 2013) | 3 |
| Alendronate70mg/wk vs raloxifen 60mg/day | 24 PM-women, RCT, 12vs12, 10 controls | 12 mths | Alendron: Tot.calc.: 2.8±0.14 Ion:1.47 Raloxif.: Tot.calc.: 2.68±0.09 Ion:1.35 Controls: Tot.calc.: 2.78±0.125 Ion:1.35 | Alendron:^a^ Tot.calc.:~2.68±0.11 Ion:1.35~ Raloxif.: Tot.calc.:~ 2.68±0.09 Ion:1.4~ Controls: Tot.calc.:~ 2.75±0.16 Ion:1.35~ | Alendr:~ 11.8 to 14.0 Raloxif:~ 16.2 to 15.0 Controls:~ 17.1 to 18.1pmol/l | 24h u-calc. excr: Alendr.: 224.6±113.03 to 245.3±118.08 Raloxif: 225.7±115.03 to 238.4±109.30 Controls: 209.0±100.68 to 311.5±162.90 mg/24h | N/A | Alendr: LS: *^vs^ + 9.0% FN: +2.1%DR: –1.6% Raloxi.: LS: *^vs^ +1.1% FN: –2.0% DR: –0.1% Contr: LS: –5.4% FN: –1.0% DR: –0.9% | N/A | (A. G., S., Y., U., & D., 2013) | 2 |
| Alendronate70 mg/wk + D-vitamin 2800IU, vs. D-vitamin 2800 IU | 30 PM-women, nphpt, random. open label. | 12 months | Treatment: 2.48±0.125 Control: 2.43±0.1 | Treatment: 2.4±0.1~ Control: 2.38±0.08~ | Treatm:~ 112±21 to 110±13 Control: ~ 106±13 to 110±14 pg/ml | 24h urine: Treatment: 185±57 to 182±62~ Control: 197±48 to 183±63~ mg/l | Baseline vs 6 mth Treatm:CTX: ↓**^vs^  OC: ↓**^vs^ Control:CTX:↔ OC:↔ | Treat:* LS: +4.7% FN: +2.6% Contr:* LS: −1.6% FN: −1.7% after 12 mths | N/A | (Cesareo et al., 2015) | 3 |
| Alendronate 10 mg/d, vs placebo | 40 PM-women, RCT | 48wk, | Alendron: 2.82 ± 0.18 Placebo: 2.81 ± 0.16 | Alendro:* 2.73± 0.04 ^(from graph)^ Placebo:~ 2.82 | Alendro: ↔ Placebo: ↔ | 24h calcium excretion: Alendro: ↓*^b^ Placebo:↔ | Alend.: ALP:↓ OC:↓ uNTX:↓  Placebo: ALP:↔OC:↔ uNTX:↔ | Alend: LS:* +3.79±4.04% FN:* +4.17 ± 6.01% DR:~ +1.01± 2.32% Placeb: LS: +0.19±2.80% FN: -0.25± 3.3% DR: +0.07± 5.50% | N/A | (Chow et al., 2003) | 2 |
| Alendronate 10 mg/d | 45 pts, 19 alen-dronate vs 26 controls, unrando-mized. | 1 year | 2.73±0.15 for whole population (8 pts with further biochemical evaluation: 2.75± 0.15, ion; 1.34± 0.07) | 8 pts: 2.58± 0.13 ion: 1.36± 0.1 | 8 pts: 109.5± 40.7 to 127.0± 33.2 pg/ml | 24h excr: 225.5 ± 211.7 to 196 ± 168.6 mg/24h | ALP:↓~, ^(signif. at 6.mth)^ Uhp:↓~ | Alend: LS:* +3.38± 0.04 FN:* +3.05 ± 0.16 DR^:n=8^ +1.15~ Contr: LS: -1.41± 0.02 FN: -0.81± 0.18 DR: N/A all % | N/A | (H. S. et al., 2001) | 3 |
| Alendronate 10 mg/d vs Placebo | 44 pts RCT, 12 mths cross-over | 1 year +1 year cross-over | Alendr.: Tot.calc.: 2.675±0.123 Ion: 1.35±0.160 Placebo: Tot. calc.: 2.64±0.138 Ion: 1.31±0.111 | Alendr.: 2.64±0.04~ ^(from graph)^ Placebo: 2.66~ | Alendr.: 17.2±3.8 to 21.1±6.0~ Placebo: 15.6±1.2 to 15.1±2.4~ | 24 h u-calcium: Alendronate: 4.97±0.7 to 3.6±0.9* mmol/d | Alend: uNTX: -66± 13.5%* ALP: -53± 7.85% * Placebo: all ↔ elevated | Alend: LS:**^vs^  +4.92% ±0.64% T-hip:**^vs^ +4.01%±0.77% FN:* +3.67%±1.63%FUD: +1.6% Placeb: all ↔ | No AE | (A.A. et al., 2004) | 2 |
| Alendronate10 mg/d | 15 pts, all treated | 2 mths | 2.675 ± 0.045 | 2.6725± 0.065~ | 114.5± 10.93 to 151.8± 22.24* pg/ml | N/A | N/A | N/A | No AE | (Makras et al., 2005) | 4 |
| Alendronate 10 mg/d vs no treatment | 32 pts, 14 treated, 18 controls (BMD lower in treatmentgroup – allocated open-label) | 2 yrs | Alendr.: 2.84 ± 0.03 Controls: 2.82 ± 0.04 | Alendr: 2.88 ±0.03 Controls: 2.69±0.04^c (from graph)^ | 105.8± 9.8. - A rise of 40 ng/l, between month 6 and 18 in treated group. | 24 h calcium excretion: Both groups: ↔ | Alend: ALP:↓*^d^ Udp:↔ OC:↓~ Uhp:↓*^d^  Contr: ALP:↔ Udp: ↔ OC:↓* Uhp:↔ | Alend: LS:**^vs^ +7.3 ± 3.1% FN:~ +2.6 ± 1.8% T-hip:~ +2.1 ± 1.8% DR:~ +0.7± 1.8% Contr: LS:* +4.0 ±1.8% FN:↓~ T-hip: ↓* DR:↓~ | 4 pts experienced dyspe-psia. | (C.R., P.J., & K.J., 2002) | 3 |
| Alendronate 10 mg/alter. Day vs no treatment | 26 elderly women random. 13 alendr. 13 no treatment | 2 yrs | Alendr: 2.75±0.1 Control: 2.725 ±0.075 | Alendr.: +0.6 ±3.0% Control: +0.2± 3.1%^e^ | Alendr.: +13±29%Control: +5.4±17%^e^ | Ca/Cr Alendr: +10±25% Control: -0.3± 27%^e^ | Alend: ALP: ↓**^vs^ OC: ↓**^vs^ Udp: ↓**^vs^ Control: ALP:↔ OC:↔ Udp:↔ | Alend: LS: ↑**^vs^ FN: ↑**^vs^ THip: ↑**^vs^ Contr: LS:↓~ FN:↓* THip:~ | N/A | (M Rossini et al., 2001) | 3 |
| ¤: albumin adjusted calcium unless stated otherwise; *p<0.05 compared to baseline; ~non-significant; *^vs^: significance compared to control-group; N/A: not available; Uhp: urinary hydroxyproline(µmol/l)GFR, ALP: alkaline phosphatase; Udp: urinary deoxypyridinoline; ICTP: C-terminal telopeptide of type I collagen; OC: osteocalcin; NTX: N-terminal telopeptide; ; CTX: C-terminal telopeptide LS: lumbar spine; T-hip: total hip; FN: femoral neck; FUD: Ultra-distal forearm; DR: distal radius.; PM-women: postmenopausal women; nphpt: normocalcaemic primary hyperparathyroidism.; £: significant decrease after 6 months, at 12 months nonsignificant; a: Reduction compared to baseline, was significant in alendronate-group after 6 mths, but insignificant after 12 mths. Alendronate reduced ionized s-calcium significantly more than raloxifen after 6 mths, but this difference too was insignificant after 12 mths.;b:significant reduction for wk 4-24, then increase to insignificant difference compared to baseline.;c: Calcium decreased and PTH increased in alendronate treated insignificantly during the first 3 mths of treatment, then returned to baseline-levels.; d: in alendronate treated patients all markers (except Udp) decreased the first year, but then rose again to a level insignificantly lower than baseline.; e: S- and U-Calcium decreased significantly in alendronate-group compared to baseline and controls during 1^st^ six months, but then slowly rose to baseline levels, PTH correspondingly rose during the first months, but then declined towards baseline-values. | | | | | | | | | | | |

Table 3: Clodronate

| Drug/ dose, g/day | Patient/ study character | Treat-ment duration | Baseline s-calc.¤, (mmol/l) | Mean s-calc, treated (mmol/l) | Mean change PTH (ng/L) | Mean change u-calc. (calcium :creatinine) | Bone turnover markers | Change BMD | Hard end-points | Study /ref. | OCEBM |
| --- | --- | --- | --- | --- | --- | --- | --- | --- | --- | --- | --- |
| Clodro-nate,IV/ IM/PO, 0.5-1.0g/d 0.1-0.2g/d 1.6-2.4g/day | 27 pts, all treated, short vs long term | 20-180 days | Short term: IV:3.16 IM: 2.98 PO: 2.82  Long term: PO: 3.15 | Short term: IV: 2.72~ IM: 2.38~ PO: 2.75~  Long term: PO: 3.10~ | Short term: IV: 480 to 690~ IM: 410 to 490~ PO:÷ Long term: PO: 400 to 413* | Excreted calcium /GFR µmol/l: Short term: IV:68 to63~ IM:83 to27* PO:62to52~ Long term: PO:65to65~ | Uhp: Short term: IV:48 to 24~ IM:76 to 38~ PO:19 to15~ Long term: PO: 48 to 26* ALP: Short term: IV: 245 to 347~ IM:225 to 227~ PO:92 to 96~ Long term: PO:147 to 95* | N/A | N/A | (Adami et al., 1990) | 3 |
| Clodronate, PO 1.6g/day | 14 pts, all treated, RCT crossover | 3mths | 2.875± 0.025 | 2.7± 0.05* | 106± 13 to 121± 15 µL eq/mL,~ | 24h: 185±29 to 113±23 mg/g creatinine* | ALP: 97±6 to 101±5~  Uhp: 37±3 to 28±2 mg/g creatinine* | N/A | N/A | (Shane, Baquiran, & Bilezikian, 1981) | 2 |
| Clodronate, PO 0.8-3.2 g/d | 30 pts (4 phpt)£ | 6 mths | N/A | ↓ a.a.-calcium | ↔ | ↓calcium: creatinine | ALP: ↓ Uhp/crea:↓ | N/A | N/A | (D.L. et al., 1980) | 4 |
| Clodronate, PO 1.0-3.2 g/d | 9 pts | 2-32wks | 2.88± 0.09 | 2.63± 0.05* after 6 wks of treatment | 1.28± 0.48 to 1.13± 0.42 µg/l~ | Fasting calc.-excr.: ↓* | Uhp:↓* ALP:↔ | N/A | N/A | (Douglas et al., 1983) | 4 |
| Clodronate^a^, 1.6-2.4 g/d vs ptx | 33 pts w. high comor-bidity, 14 clodron. 19 ptx | Mean follow up 35.5 mths | Clodro: 2.93± 0.18 PTX: 3.0 ± 0.2 | Clodro:N/A PTX: 2·45 ± 0·15 | Clodr: N/A PTX: 182·0 ± 150·2 to 21·0 ± 24·4 ng/l | N/A | N/A | N/A | Loss of renal func-tion in bis-phos-pho-nate-group (50%vs.0%)* Improvement in survival, ASA and NYHA class in PTX-pts* | (Fang et al., 2008) | 4 |
| Clodronate, 0.8-1.6g/d PO/ 300mg IV | 44 pts; 20phpt^b^, all treated | 3 months | 2.98± 0.05 | At nadir(1mth) 2.76±0.05* (Rose hereafter in majority mean remained elevated throughout treatment) | 7 pts: 303± 177 to 357± 277~ pmol/l | Fasting u-calc./creat: nadir(1mth): from 0.55±0.05 to 0.30±0.05* mol/mol cr | 12 pts: Uhp:45±11 to 26±5*  ALP: 155±129 to 138±90 IU/l~ | N/A | N/A | (Hamdy et al., 1987) | 4 |
| ¤: albumin adjusted calcium unless stated otherwise; *p<0.05, compared with baseline; ~:nonsignificant; N/A: not available; Uhp: urinary hydroxyproline, ALP: alkaline phosphatase; £:data only given for patients with phpt; a: Pamidronate IV, 60mg stat, was used in case of hypercalcaemic crisis, but clodronate was used for the long term treatment.; b:all result given in this table is for the patients with phpt. | | | | | | | | | | | |

Table 4: Mixed bisphosphonates.

| Drug/dose, mg/day | Patient/ study charac-teristics | Length of treat-ment | Baseline s-calcium¤, (mmol/l) | Mean s-calcium, treated (mmol/l) | Mean change PTH (ng/L) | Mean change u-calc. (calcium :creatinine) | Bone turnover markers | Change BMD | Hard end-points | Study/ ref. | OCEBM |
| --- | --- | --- | --- | --- | --- | --- | --- | --- | --- | --- | --- |
| Etidronate 200mg intermittent 2wk, PO 10wk pause | 22pts RCT 9 etidr. vs 13 PTX | 1 year | Etidr.: 2.7±0.125 PTX: 2.75±0.175 | Nadir: Etidr: 2.6±0.075~ PTX: 2.35±0.075**^vs^ | Etid:~ 90.5± 47.8 to 113.6±56.1 PTX: 97.6± 43.5 to 19.1± 17.9 **^vs^ pg/ml | Etidr:~ 0.38±0.15 to 0.34±0.14 PTX: 0.25±0.21 to 0.26±0.26 | 1year reduction: Etidronate: *^vs^ ALP(78%)* Upyd(91%)* Udpd(68%)* OC (67%)* PTX: ALP(46%)* Upyd(64%)* Udpd(49%)* OC (30%)* | LS: Etidr: +10%* PTX: +20% * T: no change | Spinal fracture: no differ-ence between groups | (T., T., J., A., & T., 2002) | 2 |
| Etidronate, 20 mg/kg/d | 6 pts, all treated | 6 mths | 2.84 | 2.7975^b^ | N/A | Calcium excress: 268.17 to 230^b^ mg/d | Uhp: 22.98 to 16.75* µmol/Gm cr | DR: ↔ | Several had transient gastrointestinal discomfort. | (R.A., W.B., C., M., & G.W., 1977) | 4 |
| Risedronate20-40 mg/d, for 1wk, PO 3wk pause | 19 pts, all treated | 65 days | 2.76±0.04 | 2.60±0.04* | 165±25 to 200±25* | ↓ fasting 2h u-calc./ creatinine | ALP: 238±21 to 188±17* | N/A | N/A | (C.A., M.D., D.J., & A., 1993) | 4 |
| Risedronate 35mg/wk vs PTX | 32 PM-women 16 PTX, 16 risedr. Prospective. | 2 yrs | Risedron: 2.52 ± 0.12 PTX: 2.73 ± 0.19 *^vs^ | Change in % at 24 mths: Risedron: -1.44% PTX: -14.85%*^vs^ | Change in % from baseline: Rise: +1.11% from 13.00 ± 4.26 PTX: -65.43 %**^vs^ from 10.79 ± 3.83 | 24 h u-Ca mmol/d: Rised: 6.17 ± 2.46 PTX: 7.81 ± 3.73 | ALP: Rise: -25.16% PTX: -51.85% *^vs^ OC: Rise: -3.93% PTX: -38.55%*^vs^ | aBMD: Rise: LS: +5.62% FN: +1.18% Thip: +1.68%PTX: LS: +3.47% FN: +6.28%Thip: +4.21% | Trab-vBMD: Rise: +0.24 % PTX: +4.67% *^vs^  Cort-vBMD: Rise: - 0.26 % PTX: +0.39% *^vsc^ | (Tournis et al., 2014) | 3 |
| Mixed unspecified bisphosphonates, long term treatment | 50 pts, retro-spect. | 5 years mean | 2.74±0.13 | 2.60±0.18* | 10.8± 5.7 to 10.3± 5.1~ | 0.70±0.22 to 0.55±0.25* | CTX: 0.30 to 0.26~ | LS:-2.5 to -2.1* FN: -2.1 to -2.2~ | No signi-ficant change in fracture-rate.^+^ | (D., S., E., & L.R., 2014) | 4 |
| Mixed bisphonates (1.2-1.6g/d PO/ 300 mg IV clodronate / 60 mg once pamidronate) | 23 pts all ptx, 6 had bisphosphonates prior to PTX. Retrospective. | 1-17 days | Bisph: 3.375 ± 0.15 *^vs^  No bisph: 2.975 ± 0.05 | 3.05 ±0.15 ~ (Only few days of treatment) | N/A | N/A | N/A | N/A | Bisphosphonates can prevent development of HBS.^a^*^vs^ | (Lee, Sheu, Tu, Kuo, & Pei, 2006) | 4 |
| Neridronate 100 mg/2mths IV+ Vit-D 400–600 IU daily | 60 PM-women, all treated (54 com-pleted) | 2 years | 2.675±0.15 | Decreased slightly but significantly the 1^st^ year, but then rose to above baseline level. (+0.2% ±0.2%) ^(from graph)^ | PTH:↑* - patients had D-vit. deficiancy. | N/A | At 6 months onwards: ALP:-28%* CTX:-49%* | LS: +6.7 ± 7.6%* FN: +2.9 ± 4.5%* Thip: 5.0 ± 3.9%* | N/A | (Maurizio Rossini et al., 2011) | 4 |
| Mixed bisphosphonates vs no treatment | 139 pts, 52 bisphosphonate37 no treatment, 50 controls (post PTX) | Up to 5 yrs | Bisphos: (n=52) 2.5 ± 0.2 No treat: (n=12) 2.4 ± 0.2 Controls: (n=30) 2.4 ± 0.7 | Bisphos: (n=52) 2.5 ± 0.2~ No treat: (n=37) 2.6 ± 0.2~ Controls: (n=50) 2.4 ± 0.0~ | Bisph: 204.6±56.9 to 112.7 ± 111.7~ No tre: 109.9 ± 74.4 to 152.5 ± 26.3~ Contr: 158.9 ± 29.1 76.1 ± 9.3* ng/l | N/A | ALP: Bispho:~ 206.6 ± 56.4 to 158.3 ± 58.5 No treat: ~ 173.2 ± 54.0 to 185.2 ± 73.4 Controls:~ 180.8 ± 61.5 to 144.0 ± 42.0 | Bisph: LS: –2.08 ± 1.4 to –2.18 ± 1.7 ~ No tre: LS: –1.80 ± 1.6 to –1.54 ± 1.2 Contro: LS: –1.99 ± 0.9 to –1.75 ± 1.2 | Bisphosphonate-treatment did not significantly improve quality of life. | (L., M., M., & S., 2014) | 4 |
| Mixed bisphosphonates (alendronate 92%, risedronate 7%, ibandronate 1%) vs observation/PTX | 6272pts 1408 Bisphosphonates, 1402 PTX, 3462 Observation Retros. | Median duration 55 mths | Median: Bisphos: 2.73 PTX: 2.78 Observ: 2.73 | N/A | N/A | N/A | N/A | >8 yrs treatm: Men (n=44/ 58): Bisph: LS:↑ Thip*^vs^+ 7.6% PTX: LS:↑ Thip:↓ Obs: LS:↑ Thip: -7.6% Women(n=196/318) Bisph: LS:*^vs^ +11.9% Thip:↓ PTX: LS:*^vs^ +6.3% Thip:↓ Obs: LS:↔ Thip: -6.6% | Fracture risk compared to non-treated: Bisph:↑ PTX:↓ | (Yeh et al., 2016) | 4 |
| ¤: albumin adjusted calcium unless stated otherwise; *p<0.05, significant difference compared to baseline; *^vs^ significant difference compared to control-group; ~: non-significant; N/A: not available; LS: lumar spine BMD; T: total BMD; THip: total hip BMD; ALP: p-alkaline phosphatase; Upyd: urinary pyridinoline; Udpd: urinary deoxypyridinoline; OC: osteocalcin; ; CTX: C-terminal telopeptide; +: Vit.D deficiency might have contributed to the bone fragility fractures.; HBS: hungry bones syndrome; a: 9/23 developed HBS post-PTX, none of the 6 patients treated with bisphosphonates were affected.; b: S- and U-Calcium generally decreased the first 5 weeks (significantly in some cases), but then rose again and was close to baseline after 6 mths treatment.; c: There was significant increase in within-group difference between baseline and 24 mths for the PTX-group in vBMD measures, but no other significant within-group differences were detected in either group. | | | | | | | | | | | |

Table 5: Cinacalcet

| Drug/dose, mg/d | Patient/ study character | Treat-ment duration | Baseline s-calcium¤, (mmol/l) | Mean s-calcium, treated (mmol/l) | Mean change PTH (ng/L) | Mean change u-calc. (calcium :creatinine) | Bone turnover markers | Change BMD | Hard end-points | Study/ ref. | OCEBM |
| --- | --- | --- | --- | --- | --- | --- | --- | --- | --- | --- | --- |
| Potassium-citrate, allopurinolw/w out Cinacalcet, 48.86 ± 30.09 mg titrated/d | 10 pts, phpt + nephro-lithiasis, random-ized, unblinded, crossover | 20 mths | 2.5 ± 0.2 | Without cinacalcet: 2.55 ± 0.235 vs. cinacalcet: 2.225 ± 0.15 **^vs^. | Baseli.: 136.3 ± 72.5; with-out *cina-calcet*: 126.5 ± 73.3 vs. with cina-calcet: 68.9 ± 38.7pg/ml**^vs^ | N/A | N/A | N/A | Stone for-mation: w.out cina-calcet: 3.2 ± 2.5 vs. w. cina-calcet 2.3 ± 2.8**^vs^ Diameter of stones:↓**^vs^ | (Brardi, Cevenini, Verdacchi, Romano, & Ponchietti, 2015) | 3 |
| Cinacalcet:30-120mg/d | 14 pts, all treated, open label | Median 12 mths | 3.05±0.075 ion: 1.55±0.13 | 2.475± 0.05 * Ion:1.33± 0.01* | 269± 48 to 223± 42 (-17%) * | 268±124 to 236±174~ | ALP:↔ sCTX:↔ OC:↔ | N/A | N/A | (Cetani et al., 2012) | 4 |
| Cinacalcet, 30-90 mg/d | 33 MEN1-patients, open-label, non-controlled | 12 mths | 2.7 ± 0.15 (for 28 pts who completed the study) Ion: 1.45 ± 0.075 | 2.45 * Ion: 1.285±0.0825* | median 146.7 to 136.5 pg/ml~ | 330.2 ± 146.6 to 245.6 ± 76.5 ~ | ALP:↔ Udp:↔ | LS:~ 0.876 ± 0.165 to 0.855 ± 0.131 T-hip:~ 0.806 ± 0.114 0.777 ± 0.126 g/cm2 | No serious AE, 15% experienced upper gastrointestinal side effects | (Giusti et al., 2016) | 4 |
| Cinacalcet, 30-120 mg/d, vs placebo | 15 MEN-1 pts, RCT, crossover, 20 sphpt pts as control (all treated) | 3 mths, washout, crossov. + 3 mths. Sphpt: 3mths treatment | MEN1: Tot.calc.: 2.875±0.05 Ion: 1.44±0.5 sphpt: Tot.calc.: 2.925± 0.125 Ion: 1.43±0.6 | MEN1: Cinaca.: Tot.calc.:* 2.375±0.1 Ion:* 1.20±0.6 Placebo: Tot.calc*^vs^ 2.875± 0.05 Ion: *^vs^ 1.44±0.5 sphpt: Cinac.: Tot.calc:* 2.375±0.1 Ion: 1.21±0.7* mEq/l | MEN1:Pre: 97.8± 18.9 Plac:~ 98.9± 21.5 Cina:* 68.5± 22.3 sphpt: Pre: 181.3±115.5 Cina:* 120.7±38.8 pg/ml | MEN1: Pre: N/A Placebo: 323±124 Cinac.:~ 378±21 sphpt: Pre: 295±109 Cinac.:~ 256±142 mg/day | MEN1: placebo vs. cina.: ALP:↓~ sphpt: pre vs. cina. ALP:↓~ | 4 pts, 12 mths treat-ment: LS:↔ | AE: 4 pts with nausea | (Filopanti et al., 2012) | 2 |
| Cinacalcet, 30-120 mg/d | 43 pts, sphpt, 23 symptomatic, 20 unsymptomatic, all treated | Median 44 mths | Symptom: 2.9 ± 0.175 Unsympt: 2.675 ^(from graph)^ | Sympt: 2.45 ±0.1 Unsympt: 2.3 Normo-calcaemia achieved: Symptom:56.5% Unsympt: 90% | Sym: ↔ Unsy: ↓* | 24h urinary calcium in both groups: ↔ | ALP in both groups: ↔ | Symp:~ LS:↑ FN:↓ Unsy:~ LS:↔ FN:↔ | N/A | (M. V. et al., 2015) | 4 |
| Cinacalcet 30-90mg/d + alendro-nate 70 mg/wk | 23 pts, retrospect. 10 treated with cin. + alendr. 13 treated w. cin. alone. | 12 mths | Cin +al: 2.775 ± 0.045 Cin: 2.75 ± 0.05 | Cin +al.:* -14 ±1.4% Cin:* -12 ±1.3% change from base-line | Cin+al: -29±8.4 * Cin: -25±5.9 % * change, no normal-ization | 24h ratio: Cin. + al.:* -23 ± 7.6 Cin: * -18 ± 5.5 % change | ALP:**^vs^ Cin+al: -41 ± 9.5 Cin:* -16 ± 4.8 %change from baseline | Cin+al: LS:**^vs^ +9.6± 1.4% FN:**^vs^ +3.9± 1.0% Cin.: ↔ | N/A | (Faggiano et al., 2011) | 4 |
| Cinacalcet + bisphos-phonate vs PTX | 34 pts, 17 cinac. 17 PTX, retro-spective | Min. 1 yr | Cinac: 2.6975± 0.1575 PTX: 2.6925 ±0.11 | Cina: 2.535± 0.1725* PTX: 2.37± 0.0875**^vs^ | Pre: Cina: 115.8 ± 85 PTX: 183.2 ± 131 Post: Cina:~ 92.8 ± 67 PTX: 55± 25 **^vs^ | N/A | N/A | Pre: Cin: femur: −2.25 Spine: −1.9 PTX: femur: −1.82 Spine: −1.94 Post: Cina: femur: −2.4 Spine: -1.45* PTX: femur: −1.69 ~*^vs^ Spine: −1.6* | N/A | (Keutgen et al., 2012) | 4 |
| Cinacalcet 60-270mg/d vs Placebo | 67 pts, RCT, 33 cinacalc, 34 placebo | 28 wks + open- label exten-sion | Cinac.: 2.9325± 0.1125 Placebo: 2.95±0.12 | Cina:**^vs^ 2.475± 0.025 ^(from graph)^ (-15.21 ±1.00%) Placebo: 2.9±0.025 (-1.66 ± 0.99%) Norma-lized: Cina: 75.8% vs Placebo: 0%*^vs^ | Change in % Cinac:* -23.80± 4.18% Plac.: -1.01± 4.05% | N/A | ALP: Cina: 85.5±4.1 to 97.5± 5.7 Placebo: 86.9± 6.3 to 95.0± 12.4 U/l | N/A | HR-QOL: no signify-cant difference be-tween placebo and cina-calcet | (Khan et al., 2015) | 2 |
| Cinacalcet, 30–180 mg | 20 patients, all treated, open-label | 1 year | 2.9325± 0.2125 | 2.55± 0.2375* | 181.9± 102.37 to 152.5± 70.16 pg/ml* | N/A | N/A | N/A | Medica-tion well tole-rated in 83.4% of treated | (I. & A., 2013) | 4 |
| Cinacalcet, 30-60 mg/d | 8 MEN1-patients, prospec-tive audit, all treated | 10–35 months with main-tenance | Median: 2.76 R: 2.62–2.91 | Median: 2.35* R: 2.13–2.54 | Median 15.85 R:4.8–36.5 to 9.7* R: 2.4–29.4 pmol/l | Median 24h u-calcium: 5.02 R:2.54–7.79 to 3.35~ R:1.98–5.6 mmol/24h | N/A | N/A | N/A | (Moyes, Monson, Chew, & Akker, 2010) | 4 |
| Cinacalcet, 60-360 mg/d, titrated | 17 pts w. intractable phpt, all treated | 80 wks | 3.2± 0.2 | 2.60±0.08* at end of titration-phase. 88% had a ≥0.25 mmol/l reduction | Mean: 243±26 to 396± 158~ Media: 267 to 173~ pg/ml | N/A | uNTX: 78.8 ± 38.5 to 168 ± 119 nM (median: 25 to 22nM) ALP: 60.4 ± 27.7 to 82.2 ± 37.0 (median;19 to 22)ng/ml^a^ | N/A | 88% had AE - none serious. HR-QOL: gene-rally im-proved in all mea-sures in more than 50% of subjects | (Marcocci et al., 2009) | 4 |
| Cinacalcet, 60-120mg/d | 70 pts, all treated until reffered to ptx. 58 patients were treated >1 mth and are included in these figures. | 1-28 mths | 2.925 ± 0.125 | 2.55 ± 0.1 * | 156 ± 42 at baseline. Mean de-crease after 6 mths treatment: 15.4%* | N/A | N/A | 14/23 pts treated > 1.5 yrs had >1SD decrease in BMD* | 27% stopped treatment due to nausea within 4 mths. Symptomatic hypocalcemia in 6% | (Norman, Lopez, & Politz, 2012) | 4 |
| Cinacalcet, 60-100 mg/d | 78 pts, RCT, 40 cinac., 38 placebo | 1 yr | Cinac.: 2.675 ± 0.125 Placebo: 2.675 ± 0.1 | Cinac:**^vs^ 2.425 ± 0.125 Placebo: 2.725 ± 0.175 Normo-calcaemia achieved in 73% of treated vs 5% in placebo-group*^vs^ | Cina: 105 ± 36 to 95 ± 34 pg/ml -7.6%* Plac.: 120 ± 54 to 127 ± 53 pg/ml +7.7%* | 24h u-ca/cr: Cinacalcet: 0.27 ± 0.10 to 0.29 ± 0.12 Placebo: 0.31 ± 0.13 to 0.27 ± 0.12 | Cina: ALP:↑*^vs^ sNTX: ↑*^vs^  uNTX: ↑*^vs^ Udp:↑~ Placebo: ALP:↔ sNTX:↔ uNTX:↔ Udp:↑~ | Change in Z-score at wk 52: Cina: LS:0.0 Thip:~ -0.01 DR:~ -0.05 Place: LS:~ +0.03 THip:~ -0.02 DR:~ -0.01 | 8 cina and 6 placebo pts with-drew because of adverse events. | (Peacock et al., 2005) | 2 |
| Cinacalcet, 60-120 mg/d | 45 pts open-label extension of the above mentioned study, 24 prior placebo, 21 prior cinacalcet | 4.5 yrs + parent study (5.5 yrs) 31 pts > 5 yrs | Prior Cinac: 2.725± 0.125 Prior plac: 2.675± 0.1, at baseline of parent-study. | Normal-ized(<2.575mmol/l) throughout the study in 74-92% of subjects* | Combined, base line: 109.8 ± 6.8 3 yrs: (n=36) 103.4 ± 9.2 ~ 4 yrs: (n=32) 88.6± 7.4* 5 yrs (n=30) 87.7±7.3* pg/ml | N/A | ALP in both groups: ↔ | Change in aBMD vs base-line(in Z-score): 5 yr: Prior plac.: +0.30 ±0.14~ Prior Cina: +0.22± 0.19~ | Treatment-related AE in 29% of patients; myalgia nausea, hypo-calcaemia. | (Peacock et al., 2009) | 4 |
| Cinacalcet, 60-360 mg/d | Pooled analysis of (Marcocci et al., 2009; Peacock et al., 2005, 2009) | Patients divided in three categories based on seve-rity of disease: 1 severe (n=35) 2 moderate (n=37) 3 Asymp- tomatic (n=15) | Category: 1: 2.95 ±0.3 2:2.75±0.18 3: 2.63 ±0.1 *^vs^ | Decrease in s-Calc at 6 months: 1: 17.1%* 2: 11.2%* 3: 12.4%* -remained decreased in all groups throughout observa-tion, between 7.6-14.6%. >70% had normaliza-tion in all groups. | Base-line: 1:163 ± 111 2:125 ± 57 3:103±38 pg/ml Was lowered(mostly not significantly) from 1 year throughout study-period. Group 3 generally lower than the other groups. | All groups at 1 yr: 24h u-Ca/Cr excr: ↔ | All groups at 1 yr:  uNTX:↔ sNTX: ↔ ALP: ↔ | In category 2: decrease at 1 yr in Z-score at the total femur* All other categorical and sites:↔ | 58% had treatment-related AE, none serious. | (Peacock et al., 2011) | 3 |
| Cinacalcet, 30-90 mg/d | 18 pts, 16 men 2 women, retro-spective, all treated | Mean: 8 mths. | 2.65 ±0.1325 Ion: 1.45 ± .07 | 2.365± 0.085* Ion: 1.26±0.06* (94/81% normal-ized) | 141 ± 78 to 108 ± 64.5* pg/ml 25% normal-ized | 24h u-calc. excretion: (n=5) 279 ± 202 to 244 ± 111~ mg/d | N/A | N/A | 2 pts had AE. 3/16 reported improvement in mood | (S.-C. S. & F.R., 2008) | 4 |
| Cinacalcet, 60-100 mg/d vs placebo | 22 pts, RCT | 15 days | Cinacalcet: 2.65 ± 0.21 Placebo: 2.60 ± 0.15 | Cinacal: 2.23±0.18 **^vs^ Placeb: 2.6 | Cina: predose -20.3% *~^vs^ Place: +1.8% - Nadir was between 2-4 hrs post dose. | 24h calc/cr: Cinacalc: -22%~^vs^ Placebo: +16% 24h excretion (+25% vs +11%)~ ^vs^ | N/A | N/A | No difference in AE between placebo and cina-calcet. | (D.M., J.P., S.A., L.C., & M.D., 2003) | 2 |
| Cinacalcet, 15-120mg/d | 100 sphpt 35 fphpt, all treated retrospect. | Median 12 mths | Sphpt: 2.90± 0.27 fphpt: 2.75 ± 0.17 *^vs^ | Sphpt:* 2.55± 0.22 fphpt: * 2.47 ±0.15  65(sphpt) vs 80 % (fphpt) became normocalcæmic. | Sphpt: 18 to 14* pmol/l (-22%) fphpt: 13 to 12~ (-8%) pmol/l | N/A | N/A | N/A | 6 pts stopped treatment due to AEs 21 had mild GI dis-comfort. 3 had mild hypocalcaemia. | (F. et al., 2013) | 4 |
| Cinacalcet, 15-240mg/d | 303 pts, all treated Prospect. Observa-tional. | 12 mths | 2.86 ±0.02 (n=275) | 2.53± 0.02* 71% became normo-calcaemic | 262.5±53.1 to 168.4± 13.1 (-12.66 %) | N/A | ALP: 110.6 ±71.6 IU/l | N/A | 26.7% had drug related AEs, 7.6% stopped treatment due to AEs. None life-threate-ning. | (Schwarz et al., 2014) | 4 |
| ¤: albumin adjusted calcium unless stated otherwise; *p<0.05, significant change from baseline; ~:non significant difference from baseline;*^vs^: significant difference between groups; N/A: not available; Udpd: urinary deoxypyridinoline; ALP: p-alkaline phosphatase; ; NTX: N-terminal telopeptide; ; CTX: C-terminal telopeptide; LS:lumbar spine; FN: femoral neck; sphpt: sporadic primary hyperparathyroidism; fphpt: familiar primary hyperparathyroidism; HRQOL: health-related quality of life; R:range; a: values for bone-metabolism markers were highly variable from one measurement to the next, and at the end of 80 wks follow-up mean ALP was 11.3± 5.3 ng/ml, and uNTX: 21.8 ± 6.6 nM – stated by the authors to be unchanged from baseline at the end of titration-phase. | | | | | | | | | | | |

Table 6: Other substances

| Drug/dose, mg/d | Patient/ study character | Treat-ment duration | Baseline s-calcium¤ (mmol/l) | Mean s-calcium, treated (mmol/l) | Mean change PTH (ng/L) | Mean change u-calc. (calcium :creatinine) | Bone turnover markers | Change BMD | Hard end-points | Study/ ref. | OCEBM |
| --- | --- | --- | --- | --- | --- | --- | --- | --- | --- | --- | --- |
| Ipriflavone, 1200mg/day PO | 9 pts, all treated | 21 days (all) 5pts for 42 days | 21d: 1.57±0.21 42d: 1.48±0.10 | 21d: ~ 1.54±0.17 42d:~ 1.47±0.12 | 21d:~ 111±35 to 109±35 42d:~ 112±32 to 98±33 | 21d: ~ 0.323±0.141 to 0.228±0.094 42d:* 0.274±0.072 to 0.201±0.060 | 21d: OC:↓* TRAP: ↑~ Uhp:↓*  42d: OC: ↓~ TRAP:↑~ Uhp:↓* | N/A | N/A | (M. G. et al., 1992) | 4 |
| ¤: albumin adjusted calcium unless stated otherwise; *p<0.05, compared to baseline; N/A: not available; | | | | | | | | | | | |
